# Parents’ and healthcare professionals’ perspectives on manual therapy in infants: A mixed-methods study

**DOI:** 10.1371/journal.pone.0283646

**Published:** 2023-04-06

**Authors:** Femke Driehuis, Annick Bakker-Jacobs, J. Bart Staal, Rob A. de Bie, Maria W. G. Nijhuis-van der Sanden, Thomas J. Hoogeboom

**Affiliations:** 1 IQ Healthcare, Radboud Institute for Health Sciences, Radboud University Medical Center, Nijmegen, The Netherlands; 2 Musculoskeletal Rehabilitation Research Group, HAN University of Applied Sciences, Nijmegen, The Netherlands; 3 Caphri Research School, Department of Epidemiology, Maastricht University, Maastricht, The Netherlands; Bangabandhu Sheikh Mujib Medical University, BANGLADESH

## Abstract

**Objectives:**

Manual therapy in infants is embedded in Dutch healthcare despite inconsistent evidence and ongoing debate about its safety and merits. This study examines decision-making in manual therapy in infants and explores parents’ and healthcare professionals’ perspectives on this treatment approach.

**Methods:**

This mixed-methods study consisted of an online survey among manual physiotherapists and paediatric physiotherapists exploring decision-making on manual therapy in infants and interprofessional collaboration. These data prompted further exploration and were combined with data collected with semi-structured interviews exploring parents’ and healthcare professionals’ perspectives. Interviews were analysed using an inductive content analysis approach.

**Results:**

607 manual physiotherapists and 388 paediatric physiotherapists completed the online survey; 45% and 95% indicated they treat infants, respectively. Collaboration was reported by 46% of manual physiotherapists and 64% of paediatric physiotherapists for postural asymmetry, positional preference, upper cervical dysfunction, excessive crying, anxiety or restlessness. Reasons to not treat or collaborate were: limited professional competence, practice policy, not perceiving added value, lack of evidence and fear of complications. Analysis of interviews with 7 parents, 9 manual physiotherapists, 7 paediatric physiotherapists, 5 paediatricians and 2 maternity nurses revealed that knowledge and beliefs, professional norms, interpersonal relation, treatment experiences and emotions of parents influenced attitudes and decision-making towards choosing for manual therapy in infants.

**Conclusion:**

Parents’ and healthcare professionals’ attitudes towards manual therapy in infants can be divided as ‘in favour’ or ‘against’. Those who experienced a good interpersonal relation with a manual physiotherapist and positive treatment outcomes reported positive attitudes. Lack of evidence, treatment experience and related knowledge, safety issues due to publications on adverse events and professional norms led to negative attitudes. Despite lacking evidence, positive treatment experiences, good interpersonal relation and parents feeling frustrated and despaired can overrule negative attitudes and directly influence the decision-making process and choosing for manual therapy treatment.

## Introduction

The use of manual therapy in the paediatric population provokes ethical challenges and discussions about its safety and merits [1–5]. Despite inconsistent findings on its effectiveness, manual therapy is performed in a broad population worldwide [6–10]. Various treatment approaches are described as manual therapy, such as spinal manual therapy, chiropractic, craniosacral therapy and osteopathy, while underlying theoretic rationales and specific treatment techniques vary [6, 8, 10, 11].

In the Netherlands, manual therapy is performed by manual physiotherapists (MTs); physiotherapists who completed a 3-year post-graduate Masters programme on manual therapy. Infants are treated by MTs who completed additional education to treat infants using gentle mobilization techniques. Most reported indications for treatment are positional preference, asymmetry and upper cervical dysfunction [12]. MTs who treat infants hypothesize that persistent positional preference is caused by underlying upper cervical dysfunction leading to reduced passive mobility of the cervical spine [12, 13]. However, previous research showed that positional preference can also decrease by natural course [14]. Other studies showed that paediatric physiotherapy in infants (<6 months of age) can positively change the course of skull deformation after 12 months of onset, but does not influence outcomes after 2 and 5.5 years [15, 16].

The Dutch clinical professional guideline for youth healthcare professionals, such as paediatricians, recommends against referral for and discourages manual therapy in infants with positional preference and skull deformation [17]. Instead referral to paediatric physiotherapy is recommended [17]. Data from a previously conducted Dutch cohort study, including 307 infants with indications of upper cervical dysfunction, demonstrated that manual therapy was initiated by parents’ choice (31%) or by referral by paediatric physiotherapists (PPTs) (30%) or paediatricians or maternity nurses (21%) [12]. In the Netherlands, PPTs are physiotherapists who completed a 3-year post-graduate Masters programme specifically on paediatric physiotherapy.

So, despite ongoing discussions, inconclusive evidence on effectiveness and recommendations against referral, referral rates are high. To date, the underlying reasons of parents, MTs, PPTs and other healthcare professionals to choose or explicitly not choose for manual therapy in infants, have not been explored. Therefore our study aimed to 1) gain insight into the use of manual therapy treatment of infants and interprofessional collaboration between MTs and PPTs, and 2) explore perspectives and attitudes of parents and healthcare professionals towards physiotherapeutic manual therapy in infants.

## Methods

### Study design

This mixed-methods study consisted of two phases. First, we conducted an online survey among MTs and PPTs to gain insight in the use of manual therapy in infants (<1 year) and children (1–18 years), and their interprofessional collaboration. We only included the data regarding infants; the data regarding children did not fall within the study’s scope, and have been published in Dutch elsewhere [18]. Second, we performed individual semi-structured interviews with parents and healthcare professionals to further explore their underlying rationale, perspectives and attitudes towards manual therapy in infants. The interview study was approved by the medical ethics committee of the Radboud university medical center (CCMO-number: 2017–3914). The research team were four researchers with a background in physiotherapy (FD, RN, TH, JBS) and one research assistant (ABJ). The qualitative part of this study is reported in accordance with the COREQ-checklist [19].

### Study procedure and participants

The online survey was part of an evaluation procedure of the Dutch Association for Manual Therapy (NVMT) and the Dutch Association for Paediatric Physical Therapy (NVFK), to get insight in the interprofessional collaboration of MTs and PPTs in daily practice. The survey’s questions were constructed by FD, RN, TH in collaboration with the NVMT and NVFK. Thirty questions focused on the use of manual therapy in infants, including indications and reasons for treatment, the referral process and interprofessional collaboration. A survey participation invitation was sent to members of the NVMT (n = 2045) and NVFK (n = 865) through digital newsletters and social media. These members reflected, respectively, 48% and 47% of all registered MTs and PPTs in the Netherlands. The results of the online survey formed the basis for the individual semi-structured interviews.

The research team (FD, RN, TH, JBS) developed an interview guide (S1 File) through consensus meetings. Open questions focused on underlying beliefs about, perspectives and perceptions on or experiences with manual therapy in infants, and explored the decision-making process to consult (for parents) or refer to or collaborate with (for healthcare professionals) an MT.

Parents were recruited through MTs who participated in a previous study [20] and through social media. MTs and PPTs who indicated to be willing to participate in follow-up research when completing the online survey, were invited to be interviewed. Healthcare professionals were recruited through general invitations to hospitals and youth healthcare institutions, and by the participating MTs and PPTs. Interested and eligible parents and healthcare professionals received an information letter with the study objectives, planning and execution of the interview. After written informed consent was obtained, an interview was scheduled. All participants had no previous (treatment) relationship with members of the research team.

## Data collection

An online survey tool (Survey Monkey, California, USA) was used to collect data from September to December 2016, and consisted of 30 multiple choice and open-ended questions. The survey was accessible for all members of the NVMT and NVFK who received an open invitation.

For the interview study, participants received a short questionnaire to obtain demographic information, such as age, gender, parents’ highest educational level, and healthcare professionals’ working experience. All interviews were conducted by the primary author (FD; female, physiotherapist, health scientist and researcher with additional education in qualitative research) by telephone or face-to-face in March 2018, and were audiotaped and transcribed verbatim. Before the interview, FD introduced the study topic and checked if participants still approved with audiotaping. The semi-structured interview guide was used and field notes were taken during each interview. This enabled us to further improve subsequent interviews and to determine saturation [21, 22]. To check whether information was understood correctly FD summarised the main perceptions of the interviewee during and at the end of the interview. When new interviews revealed no new information, saturation was achieved and recruitment of participants stopped [23].

### Data analysis

**Fig 1:**
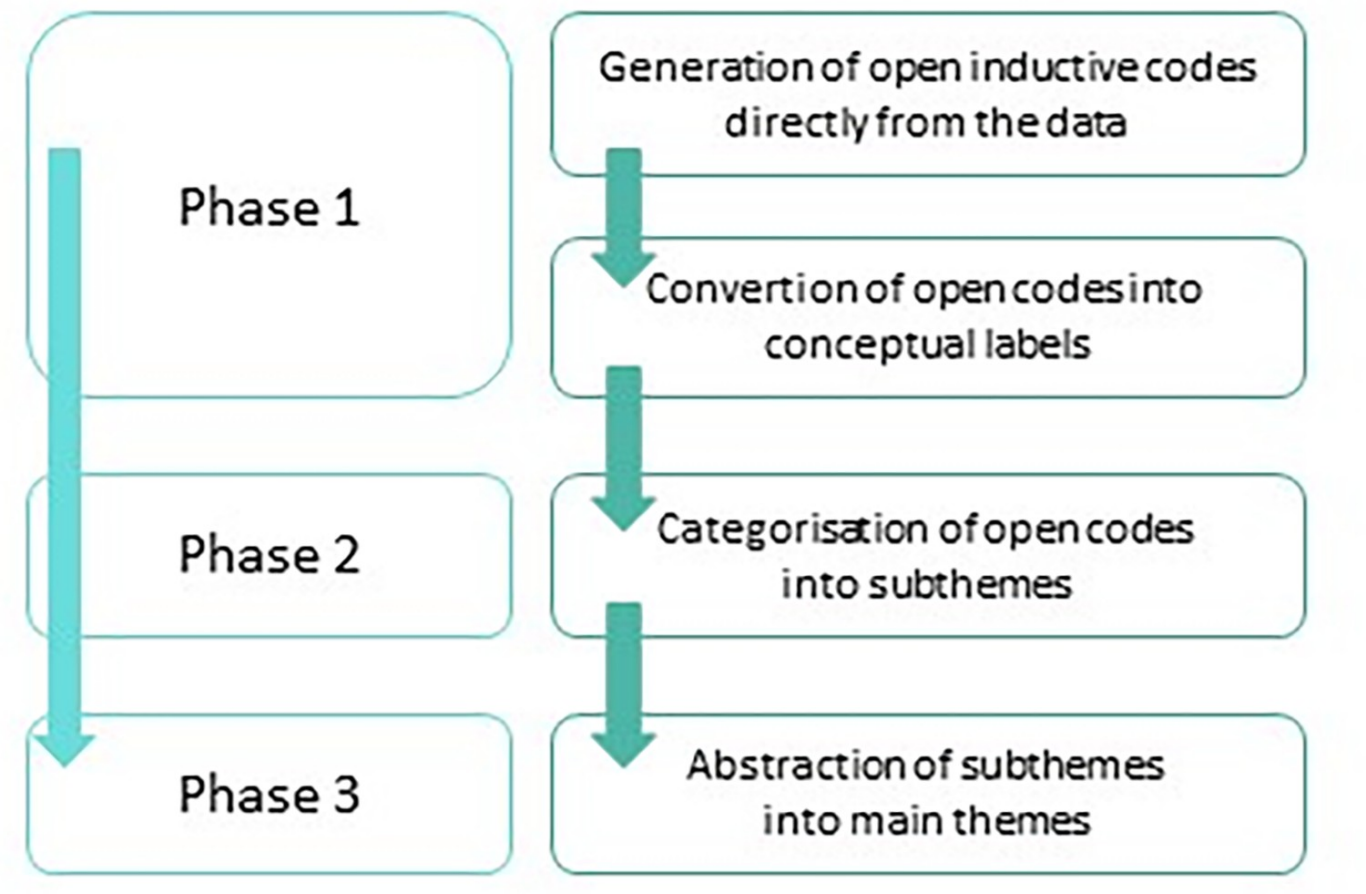
Phases of inductive content analysis.

Data of the online survey were anonymised prior to analysis. Only respondents who indicated to be willing to participate in future research on this topic shared their e-mail address with the research team. These e-mail addresses were separately stored and thereafter deleted leading to fully anonymised data. Quantitative data were analysed using descriptive statistics with SPSS Statistics, v.22.0. The qualitative data in the open questions of the online survey were coded into categories and thematically ordered by FD which enabled data quantification. Interview data were analysed using inductive content analysis and contained three phases: 1) generating open inductive codes directly from the data leading to conceptual labels, 2) categorizing open codes in subthemes, and 3) abstracting subthemes into main themes (Fig 1) [21, 22].

The initial ten interviews were coded independently by two authors (FD, ABJ), using Atlas.ti Software v.8.4.15 (ATLAS.ti Scientific Software Development GmbH, Berlin, Germany). Codes were discussed until consensus was reached. Thereafter, the remaining interviews were coded by FD, and new emerging codes were discussed with ABJ. In two consensus meetings the research team discussed the codebook, identified and redefined categories, subthemes, and themes, and discussed the relation between subthemes and themes. Within each theme, we focused on similarities and differences between parents and healthcare professionals, and between subgroups of healthcare professionals.

## Results

### Online survey: The use of manual therapy and interprofessional collaboration

A total of 607 MTs (30% response rate) and 388 PPTs (45% response rate) completed the online survey. Of the MTs, 45% (n = 272) indicated to treat infants in clinical practice, of which 28% (n = 77) treated more than five infants per month. Forty-six percent (n = 125) treated infants in collaboration with PPTs; 45% of these 125 MTs collaborated with PPTs in >75% of all infants they treat. Nearly all PPTs (95% (n = 368)) treated infants; the majority (78%, n = 286) treated more than five infants per month. Sixty-four percent (n = 237) collaborated with MTs, of which 85% collaborated in <25% of all infants they treat. Indications for interprofessional collaboration were postural asymmetry, positional preference, plagiocephaly and/or upper cervical dysfunction, and parent-reported excessive crying and anxiety/restlessness. MTs reported that collaboration was mostly initiated jointly (37%) or by MTs (34%). According to PPTs, the initiative was taken mainly by PPTs (62%) (Table 1). Both MTs and PPTs reported that collaboration was most common in infants younger than 6 months of age.

**Table 1:** The use of manual therapy in infants and interprofessional collaboration between manual physiotherapists and paediatric physiotherapists.

**Use of manual therapy in infants**		
	**MTs (n = 607)**	**PPTs (n = 388)**
**Treatment of infants (yes)**	272 (45%)	368 (95%)
Indication of number of treated infants per month		
• *<5 per month*	195 (72%)	82 (22%)
• *≥5 per month*	77 (28%)	286 (78%)
**Interprofessional collaboration**		
	**MTs (n = 272)**	**PPTs (n = 368)**
**Interprofessional collaboration in treatment (yes)**	125 (46%)	237 (64%)
Frequency of interprofessional collaboration		
• *<25% of infants*	43 (34%)	201 (85%)
• *25–50% of infants*	14 (11%)	19 (8%)
• *50–75% of infants*	12 (10%)	12 (5%)
• *>75% of infants*	56 (45%)	5 (2%)
	**MTs (n = 125)**	**PPTs (n = 237)**
**Initiating healthcare professional**		
• Manual therapist	42 (34%)	4 (2%)
• Paediatric physiotherapist	29 (23%)	146 (62%)
• Manual therapist and paediatric physiotherapist together	46 (37%)	38 (16%)
• Parents	3 (2%)	12 (5%)
• General practitioner/paediatrician	1 (1%)	5 (2%)
Indications for collaboration*		
• Excessive crying	67 (54%)	152 (64%)
• Restless/anxious	52 (42%)	135 (60%)
• Feeding problems	38 (31%)	70 (30%)
• Asymmetry	79 (63%)	158 (67%)
• Positional preference	93 (74%)	170 (72%)
• Plagiocephaly	69 (55%)	162 (68%)
• Upper cervical dysfunction	88 (70%)	103 (43%)
• Not willing to lay in prone position	53 (42%)	135 (57%)
• Other**	23 (18%)	12 (5%)

MTs: manual physiotherapists, PPTs: paediatric physiotherapists

*Multiple answers were possible; **Other indications were problems in motor development, sleeping problems or problems with posture.

The other MTs (n = 147) and PPTs (n = 131) reported no collaboration for various reasons. MTs lacked contacts with PPTs (33%), were not interested in collaboration (33%) or were limited by the practice policy (33%). PPTs reported lack of added value (31%) and evidence (17%), perceived no treatment indication (16%), no personal contacts (11%), lack of knowledge and expertise (9%), collaboration with osteopaths (9%) and fear of complications (7%). Those MTs (n = 335) and PPTs (n = 20) who reported to not treat infants at all, reported lack of professional competence (in terms of knowledge and skills) and infants not being their target population as main reasons. Additionally MTs feared complications, perceived no treatment indication, lacked experience or deemed it not evidence-based (S1 Table).

### Interview study: Perspectives and attitudes

Nine parents and 75 healthcare professionals were interested to participate in our interview study. Seven parents were included; two parents were ineligible because they visited an osteopath instead of an MT. We included 23 healthcare professionals: 9 MTs, 7 PPTs, 5 paediatricians and 2 maternity nurses. After 30 semi-structured individual interviews with parents and healthcare professionals, saturation was achieved as no new information emerged from the data. Overall, 15 participants had experience with manual therapy in infants and 15 participants did not. The interviewees’ characteristics are outlined in Table 2. Interviews were conducted by telephone (n = 27) and face-to-face (n = 3). The interviews took between 15 and 35 minutes (mean: 24, SD: 6).

**Table 2:** Characteristics of interviewees (n = 30).

**Parents (n = 7)**					
**Interviewee**	**Gender**	**Age (years)**	Experience*	**Highest level of education**	
P01	Male	32	No	Higher education/University	
P02	Female	30	Yes	Higher education	
P03	Female	31	Yes	Higher education	
P04	Male	35	No	Higher education/University	
P05	Female	29	Yes	Higher education	
P06	Female	34	No	Higher education/University	
P07	Female	32	Yes	Higher education/University	
**Healthcare professionals (n = 23)**					
**Interviewee**	**Gender**	**Age (years)**	Experience*	**Profession**	**Work experience (years)**
H01	Female	61	Yes	MT	40
H02	Male	59	No	MT	39
H03	Female	43	Yes	PPT	22
H04	Female	60	Yes	PPT	35
H05	Male	56	No	MT	31
H06	Female	40	Yes	PPT	15
H07	Female	35	No	PPT	10
H08	Female	51	Yes	MT	23
H09	Male	42	No	MT	18
H10	Male	52	Yes	MT	22
H11	Female	59	Yes	MT	17
H12	Female	28	Yes	PPT	3
H13	Male	59	Yes	MT	16
H14	Female	29	No	PPT	3
H15	Female	36	No	PPT	10
H16	Female	52	No	PED	21
H17	Female	53	Yes	PED	26
H18	Male	42	No	MT	4
H19	Female	39	No	PED	13
H20	Female	43	Yes	Nurse	15
H21	Female	59	No	Nurse	10
H22	Female	33	No	PED	6
H23	Female	48	Yes	PED	12

*Experience with manual therapy in infants (e.g. visit, referral, collaboration); MT: manual physiotherapist, PPT: paediatric physiotherapist, PED: paediatrician

In line with the outcomes of the online survey, the interviewees with experience with manual therapy reported signs of asymmetry, persistent positional preference, excessive crying behaviour and reduced cervical mobility as treatment indications for infants.

In the exploring of perspectives and attitudes, we identified five main themes and 12 subthemes. The main themes were knowledge and beliefs, professional norms, interpersonal relation, treatment experiences, and emotions.

In the sections below, we described the results per subgroup to highlight differences and similarities: outcomes of parents and healthcare professionals are reported separately and within the subgroup of healthcare professionals we distinguished between MTs, PPTs and paediatricians/maternity nurses if relevant. Another distinction was made between participants with experience (exp) with manual therapy in infants (i.e. seek treatment, referred or treated), and those without experience (no-exp). Per subtheme illustrative quotes were added in the text and quotes per specific subgroup are presented in Table 3.

**Table 3:** Quotations of participants reported per theme and subtheme*.

**Theme 1: Knowledge and beliefs**	
***Subtheme 1*: *Scope and purpose of manual therapy***	
“Actually I don’t know much about it, except that I relate it to ‘cracking’ the spine. Or at least, I guess that’s what manual therapy is. I do it myself as well, because when I had lower back problems, I visited a manual therapist who did this as well. Infants are so vulnerable, I won’t let them do that with my child”	Parent, no-exp [P04]
“I would like to know how many babies have this problem and what manual therapy could do to solve it. Also, I prefer information on what manual therapy is and does. For me personally, information about potential risks is also important. On online discussion platforms you read a lot as a parents, without knowing what is true. Honestly, I think I don’t have a realistic picture of what manual therapy entails.”	Parent, no-exp [P01]
“I have talked to paediatricians and they say that they don’t know what physiotherapy or manual therapy can do and what it contains. They think manual therapists only manipulate the spine, using lots of power. But they have never explored other treatment options in children with positional preference and excessive crying behaviour. So actually, they just don’t know.”	MT, exp [H10]
“Infants seem to experience distress, they cry a lot and are restless. They react very strongly when I carefully hold and turn their neck. They show reduced cervical mobility. As a manual therapist, I can correct this and relatively quickly increase mobility.”	MT, exp [H13]
“People often don’t know what manual therapy is or can do. A lot of the parents I meet think it’s dangerous. But actually, manual therapy treatment is so different than they think. They have no idea”	PPT, exp [H12]
*“I actually don’t know what manual therapists do in infants*. *I think a lot of parents don’t know this either*. *Personally*, *I would like to have information about what they do and why*.*”*	Nurse, no-exp [H21]
“As a nurse I cannot refer directly to manual therapy. According to our guideline, infants have to be referred to the paediatric physiotherapist. When they assess the infant’s cervical spine, they notice a deviation, a dysfunction. Manual therapy can resolve that, with mobilizations. I have seen that once.”	Nurse, exp [H20]
“As a doctor, I would like to inform parents when they have questions about treatment options. Now I tell them that I don’t know enough about manual therapy to help them. This doesn’t feel good to me. It would help if manual therapists could define what they do, for what indications and why it would help.”	Paediatrician, no-exp [H19]
***Subtheme 2*: *Beliefs of added value***	
“I felt confident that the positional preference would disappear sometime, spontaneously”.	Parent, no-exp [P06]
“I think I am of great value for the asymmetric infant; a lot of other paediatric manual therapists as well I assume. Because parents experience quick results after treatment, my work is very rewarding.”	MT, exp [H13]
“With paediatric physiotherapy you can achieve so much in infants. Sometimes it might take a little longer, but treatment outcomes are always good. With advice and instructions for parents on active exercises they can do with their child at home, positional preference or asymmetry of the head can be corrected. I think paediatric physiotherapy is enough.”	PPT, no-exp [H15]
*“I hope for more scientific research on effectiveness*. *If that shows that manual therapy can help these infants and parents*, *I am okay with that*. *Then I prefer parents to visit a manual therapist instead of letting their child take all kinds of medication and stuff*.*”*	*Paediatrician*, *no-exp [H19]*
***Subtheme 3*: *Safety issues***	
“I imagine that they [manual therapists] are going to ‘crack’ the spine of my child. That scares me. That is not okay.”	Parent, no-exp [P04]
“In terms of high quality healthcare, safety is really important. So, if we cannot say that a particular treatment is effective and we don’t know whether it can potentially cause damage or increase other risks, I think you should just not do it until there is more evidence.”	MT, no-exp [H09]
“One time I saw, I think a manual therapist, on internet treating an infant. I have also heard stories about that they pull and turn the infant’s neck and sometimes nearly fold the child. Look, alternative therapies can be okay if it helps people, but this…this scares me. The infant’s neck is so vulnerable, such a little baby. No no!”	*Nurse*, *no-exp [H21]*
*“Well*, *in general I hear positive experiences of parents*. *But to me it is terrifying*. *I will never do it or advice parents to go*. *I do give my opinion to parents; not that I think it is bad*, *but that I personally would never go*.*”*	*Nurse*, *no-exp [H21]*
***Subtheme 4*: *Media publicity***	
*“It just scared me*. *The first things I read online were ‘baby died after manual therapy’*, *‘therapist folded child in half’*, *that kind of things*. *That influenced me*. *I thought ‘No no*, *I am not gonna do this*.*”*	*Parent*, *no-exp [P01]*
“The paediatricians in one hospital we work with are really positive, doctors in another hospital are definitely not. The reason is simple; a few years ago a child died after treatment. The unfortunate thing is that treatment was performed by a therapist who was not a paediatric manual therapist or educated to perform treatment. That went completely wrong! Horrible. But that kind of story goes around and from a certain moment people say that manual therapy in infants is dangerous and should not be performed. That is hard sometimes, because it was not manual therapy which was performed. Parents also hear these stories. So I have to explain everything very carefully to gain their trust in me and the treatment.”	MT, exp [H11]
“But an actual manipulation of the spine in an infant, who cannot communicate and say when to stop…no, no, that is dangerous to me. Unfortunately, in the past this had also led to complications. One child died after certain therapy!”	Paediatrician, no-exp [H19]
**Theme 2: Professional norms**	
***Subtheme 5*: *Professional standard***	
“The most important reason why I don’t treat infants, is that I think infants are not the domain or target group of manual therapists. The professional standard of manual therapists says nothing about children. In the post-graduate Masters programme Manual Therapy there is little to no attention for children. You have to know more about the paediatric development, and that belongs more to the domain of paediatric physiotherapists.”	MT, no-exp [H09]
*“In terms of development*, *motor control and parental coaching*, *yes*, *that is obviously the paediatric physiotherapeutic domain*. *But problems related to spinal dysfunction belongs to the manual therapeutic domain*. *We can reduce the cervical dysfunction in infants pretty quick*. *As mobility has increased*, *treatment goals are easier and quicker to achieve for the paediatric physiotherapist*. *In my opinion*, *manual therapy is supplementary to instead of a substitute for paediatric physiotherapy*.*”*	*MT*, *exp [H13]*
“I think children belong to paediatric physiotherapists. We are specifically educated in the development of children and know more about their pathology and symptoms. Manual therapists only focus on the neck, but it is about the bigger picture of the child.”	PPT, no-exp [H04]
***Subtheme 6*: *Guideline adherence***	
“Because of the guideline, doctors [in youth healthcare] unfortunately do not advice for manual therapy. I know that these doctors adhere to these guideline. It’s their professional basis. And I think it’s hard for them to ignore the guidelines and advice parents different than what is described in the guideline.”	MT, exp [H10]
“Doctors [in youth healthcare] always refer for paediatric physiotherapy. We frequently have meetings with them. They know that we work together with manual therapists. They also see the positive outcomes. But they refer to us, so that we can refer to the manual therapist, alongside our own physiotherapeutic treatment. I would call it an indirect referral.”	PPT, exp [H12]
“I think it’s a pity that manual therapy is completely ignored within youth healthcare. That is mainly because of the guideline. I have to adhere to that. But, honestly, I think it’s more about the experience people have with manual therapy. If they do, like when they know a therapist personally or visited their practice once, they know more about what they do. I know that those colleagues ‘secretly’ refer people for manual therapy. Officially, according to the guideline, we have to discourage parents to go.”	Paediatrician, exp [H17]
*“You know*, *I don’t refer directly*, *because of those experiences in the past [publications of adverse events]*, *and because there is too little evidence*. *So*, *I don’t do it directly*. *But I do always say*, *it’s fine by me if parents go themselves*. *I won’t advice negatively*. *I hear lots of positive experiences from parents*, *that they see results directly*.*”*	*Paediatrician*, *exp [H16]*
**Theme 3: Interpersonal relation**	
***Subtheme 7*: *Professional relationship***	
“She [manual therapist] was a colleague of my sister. So I knew she was good. That is why I trusted her.”	Parent, exp [P02]
“I think you should think the same about these complaints and symptoms and clinical reasoning, to have a good professional relationship and collaboration. If it is missing, collaboration doesn’t work.”	MT, exp [H05]
*“To me communication is really important*. *When I refer a child*, *I would like to hear back how the treatment is going and what has been done*. *Open communication*, *where we respect each other*, *is really important for collaboration*.*”*	*PPT*, *exp [H23]*
“I only refer to people that I know personally.”	Paediatrician, exp [H17]
***Subtheme 8*: *Professional expertise***	
“Of course you always feel a little nervous when someone else is handling your baby. Especially because of the neck and the vulnerability of a baby. But she [manual therapist] really explained everything so well. She seemed very calm and professional, that made me trust her. Also because she talked us through the treatment session, she explained what she did and why. That really helped.”	Parent, exp [P03]
“My sister is a physiotherapist and told us about manual therapy. We mentioned this in a consult with the youth healthcare professional. We heard mixed messages about it, so we wanted to be sure. She told us that if we could find a registered manual therapist, we could go.”	Parent, exp [P02]
“I think it is important to understand that treating infants is a specialty within manual therapy. You should not do it when you are not properly educated and trained.”	MT, exp [H18]
*“To me it is just really important that it [a manual therapist] is someone that I can trust*, *that he knows what he is doing and that I have seen him working*. *I was with him once with my own son*. *So I know what he does and how professional he is*. *I would never refer to a paediatric manual therapist who I don’t personally know*.*”*	*PPT*, *exp [H03]*
“I have good experiences with manual therapists. Sometimes I refer a child to them. I trust them, and their expertise; that they treat the child gently and treatment will be performed safely.”	Paediatrician, exp [H16]
**Theme 4: Treatment experiences**	
***Subtheme 9*: *Personal experiences***	
*“Actually*, *it [treatment] helped very well with our daughter*, *really*. *Because we did not wait too long*, *symptoms were quite easy to resolve*. *Our therapist gave us confidence in what the treatment entailed and how it was performed*. *So*, *yes*, *our daughter really did benefit from it*. *I think treatment can also prevent future problems and complaints*. *When really something is not functioning well in the neck*, *well*, *yeah*, *that doesn’t resolve itself*.*”*	*Parent*, *exp [P03]*
“I am glad we went for therapy. People around us told us already it [manual therapy] was good. But yeah, we can see that now. The son of our neighbours also had a strong positional preference of his head, they did not go and now that little boy has a flat head.”	Parent, exp [P02]
“Well, in my experience, the outcomes of treatment are spectacular within a few sessions. Infants don’t have a positional preference anymore; the mobility has really improved. A lot of times parents say they got ‘a different child’ after treatment, because their child sleeps and eats better and cries less.”	MT, exp [H13]
“Parents read about manual therapy online or have heard about it from friends. I never actively refer for manual therapy. I do explain to parents what manual therapy can do. It is the decision of the parents themselves. I don’t wanna hear later ‘Why did you never tell us about manual therapy?’. I inform them, but it’s the parents’ choice to go. That’s not my responsibility”	PPT, no-exp [H04]
“I want to resolve the infant’s problems with my own paediatric physiotherapeutic tools first. Sometimes I decide, together with parents, to refer for manual therapy. In my experience the manual therapist resolves the problem in 9 out the 10 cases, and actually always very quickly as well.”	PPT, exp [H06]
“I inform them [parents] about physiotherapy. Sometimes parents ask what I think of manual therapy. Then I tell them it’s not my advice and infants are so vulnerable and small. But I let parents decide to go or not. I do always tell them to pay attention what the therapist does. If they don’t feel comfortable with that, they should stop. I always want to have that said. Other than that, it’s the parents’ choice.”	Paediatrician, no-exp [H22]
*“I think it’s a pity that manual therapy is completely ignored within youth healthcare*. *That is mainly because of the guideline*. *I have to adhere to that*. *But*, *honestly*, *I think it’s more about experience people have with manual therapy*. *If they do*, *like when they know a therapist personally or visited their practice once*, *they know more about what they do*. *I know that those colleagues ‘secretly’ refer people for manual therapy*. *Officially*, *according to the guideline*, *we have to discourage parents to go*.*”*	*Paediatrician*, *exp [H17]*
***Subtheme 10*: *Experiences from the social environment***	
“I have heard a few colleagues and friends talking about it [manual therapy]. They tell each other, you know, those young parents. Most of the time it worked for them, and they say others should go too.”	Parent, no-exp [P01]
“People told me I should quickly go to a manual therapist. I didn’t know much about it, so it surprised me. But a lot of people told me, so we went. And it also really helped, so I am glad we did go.”	Parent, exp [P03]
“Because we heard in our social environment of it [manual therapy] and that it should help, because otherwise the head of the child will be malformed. So yeah, we thought that we could always just try it. We don’t want a child with a flat head.”	Parent, exp [P05]
*“Parents want to fix their child*, *so they keep ‘shopping for healthcare’*. *But in my opinion that’s not how the healthcare system should work*, *that costs the society a lot of money*. *But I see in parents that experience fear that they believe something might be wrong*, *and feel*, *yes*, *how to say it*, *social pressure to seek for help*. *Parents tell each other they want the best for their child*, *really*, *they kinda push each other*.*”*	*MT*, *no-exp [H05]*
“Most of the time parents are referred by youth healthcare professionals. They know my name. But I also see parents who have visited me before or heard stories from other parents. These young parents know how to find each other. And social media also plays a role.”	MT, exp [H13]
“Parents ask me about it [manual therapy]. They have heard it from other parents.”	Paediatrician, exp [H17]
**Theme 5: Emotions**	
***Subtheme 11*: *Feelings of frustration and desperation***	
*“Normally*, *I am not a contemporary and alternative therapy type*. *But now I was so desperate*. *I didn’t know what to do anymore*. *I couldn’t handle it anymore*. *So I just went [to manual therapy]*. *The crying is now better*, *but I doubt if it’s because of therapy or just natural course*. *That’s hard to say*.*”*	*Parent*, *exp [P07]*
“We [parents] saw that he [child] was in a lot of pain. We felt so helpless. Because, well yes, he can’t tell what’s going on with him, and he cried so excessively. And then you come to a point that you keep looking for something that could help. We just wanted to try everything that might help him. And then we read information about manual therapy and then we recognised all the symptoms. They actually described our child. We felt so relieved.”	Parent, exp [P02]
“Parents can get very emotional. They want the best for their child; try the best they can to incorporate advices from youth healthcare or paediatric physiotherapists in daily life and do exercises with their child. But sometimes it doesn’t work. And that really frustrates them.”	MT, exp [H08]
“A lot of the times I see parents who are desperately looking for a solution. I think that as a healthcare professional you should be aware of this, and look for options with them which are realistic. So that also means you sometimes have to say to these parents “This is a period every child goes through, it will be fine”. In lot of cases, it is just the natural course for these babies. And when parents know this, they feel less stressed.”	Paediatrician, no-exp [H19]
***Subtheme 12*: *Feelings of concern***	
“As a parent you are emotionally involved with your child. You see things differently, even when you are a healthcare professional who values evidence yourself. It could happen that all of a sudden evidence is not that important anymore and you think; ‘let’s just try it, you never know, maybe it does help’.”	Parent, no-exp [P01]
“We [parents] heard from people around us that it [manual therapy] could help. So yeah, we thought ‘if it doesn’t help, it also doesn’t hurt’. Let’s give it a try”	Parent, exp [P07]
*“I think that young parents can be really insecure and concerned; that makes them vulnerable*. *Their tendency to seek help and see every doctor or therapist because it might help*, *to stop the crying*, *is really strong*.*”*	*PPT*, *exp [H04]*

*Quotes in italics are also incorporated in the Results section; MT: manual physiotherapist, PPT: paediatric physiotherapist; where interviewees mentioned ‘manual therapist’ it refers to a manual physiotherapist.

### Theme 1: Knowledge and beliefs

#### Scope and purpose of manual therapy

Parents and healthcare professionals with manual therapy experience (exp) mentioned they know the aims of treatment and what treatment entails. Parents, PPTs, paediatricians and nurses without experience (no-exp) mentioned they lacked knowledge about the scope, purpose, and indications for treatment, and what treatment entails. They indicated that it was hard for them to distinguish between manual therapy, chiropractic, and osteopathy and associated manual therapy with the ‘cracking-the-spine’ treatment in adults. Parents (no-exp) pointed out they felt the need to know how manual therapy works and how it could help their child, before considering treatment. PPTs and paediatricians (no-exp) stated that because of their lack of knowledge they did not advise parents to go for manual therapy.

*“I actually don’t know what MTs do in infants*. *I think a lot of parents don’t know this either*. *Personally*, *I would like to have information about what they do and why*.*” (H21*,*no-exp*)

#### Beliefs of added value

Healthcare professionals (no-exp) mentioned that they believe there is no added value of manual therapy in infants due to lack of evidence for this treatment and their positive experiences with paediatric physiotherapy as a sole treatment approach. They indicated that if there was more evidence supporting the use of manual therapy in infants, they might consider it. On the other hand, other healthcare professionals (exp) and parents (exp) perceived an added value of manual therapy because of its ‘hands-on’ treatment of the infant’s cervical spine in comparison with the more ‘hands-off’ paediatric physiotherapeutic treatment. They believed that a dysfunction in the cervical spine can lead to asymmetry and reduced mobility.

*“I hope for more scientific research on effectiveness*. *If that shows that manual therapy can help […]*, *I am okay with that*.*” (H19*,*no-exp*)

#### Safety issues

Parents and healthcare professionals (no-exp) felt there is lack of information about potential treatment risks, which negatively influenced their attitude towards manual therapy. According to parents, paediatricians and nurses (no-exp), manual therapy was associated with high-velocity thrust manipulation of the spine. They pointed out that because of the vulnerability of infants in general they described manual therapy as risky. Parents (no-exp) mentioned they believe MTs push and pull the infant’s neck, which scared them. Paediatricians (no-exp) believed that treatment should only contain gentle mobilizations and no manipulations. Parents and healthcare professionals with experience (exp) indicated manual therapy as a safe treatment technique because they knew MTs performed only gentle mobilizations of the spine.

*“To me it is terrifying*. *I will never do it or advise parents to go*. *I do give my opinion to parents; not that I think it is bad*, *but that I*, *personally*, *would never go*.*” (H21*,*no-exp*)

#### Media publicity

Several years ago two separate case-reports describing an infant’s death after spinal manipulation by respectively a craniosacral therapist and Vojta physiotherapist [7, 24] led to discussion and media publicity. Healthcare professionals (no-exp) brought up that these publications made them more aware of potential risks and more hesitant towards manual therapy. Parents (no-exp) expressed that they read multiple stories about these two events on social media and online discussion platforms and this scared them. MTs (exp) stated that these publications negatively impacted parents’ and healthcare professionals’ view on manual therapy in infants and subsequently interprofessional collaboration. They felt they constantly have to thoroughly explain what their treatment entails, and how it differs from the treatments described in those publications.

*“It just scared me*. *The first things I read online were ‘baby died after manual therapy’ […]*. *I thought ‘No no*, *I am not going to do this*.*” (P01*,*no-exp*)

### Theme 2: Professional norms

#### Professional standard

Healthcare professionals with and without experience in manual therapy in infants expressed differing views on whether infants belong to the manual therapeutic domain. Some MTs and PPTs (no-exp) stated that, according to their professional standards, children—and specifically infants–solely belong to the paediatric physiotherapeutic domain. They highlighted that PPTs have extensive knowledge about paediatric development and specific pathology. According to other MTs (exp), infants do belong to MTs’ professional domain once MTs have completed additional education in paediatric manual therapy. Both groups (exp and no-exp) experienced lack of clarity on the vision and policy of their national professional associations.

*“In terms of development*, *motor control*, *and parental coaching*, *yes*, *that is obviously the paediatric physiotherapeutic domain*. *But problems related to spinal dysfunction belong to the manual therapeutic domain*. *[…] Manual therapy is supplementary to instead of a substitute for paediatric physiotherapy*.*” (H13*,*exp*)

#### Guideline adherence

The current clinical practice guideline for youth healthcare professionals recommends paediatric physiotherapy in infants with positional preference and/or skull deformation and discourages referral for manual therapy based on publications on adverse events in infants after spinal manipulation [17]. Paediatricians (no-exp) reported adherence to this guideline as their main reason for non-referral. However, other paediatricians (exp) explained that experiences of positive treatment outcomes could lead to referral and therefore poor guideline adherence. MTs (exp) mentioned they experienced resistance towards referral and the treatment itself from paediatricians because of this guideline.

*So*, *I don’t do it [referring] directly*. *[…] it’s fine by me if parents go themselves*. *I won’t advice negatively*. *I hear lots of positive experiences from parents*.*”(H16*,*no-exp*)

### Theme 3: Interpersonal relation

In this study, an interpersonal relation refers to the relationship between MTs and parents or MTs and other healthcare professionals, on an individual level. Parents and healthcare professionals (exp and no-exp) mentioned that the feeling of trust in a particular MT’s professional capabilities and expertise influenced their attitude, but could also directly influence decision-making and choosing for manual therapy. Healthcare professionals (exp) revealed that a good interpersonal relation can overrule negative attitudes based on professional norms and directly affects the decision-making process. The perceived interpersonal relation with MTs, and thereby trust, was influenced by two factors: professional relationship and professional expertise.

#### Professional relationship

According to parents and healthcare professionals (exp), the relationship they had with an MT was based on personal contact. PPTs and paediatricians (exp) mentioned that if they knew an MT personally, they considered him/her as reliable and professional, leading to referral. Interdisciplinary communication, characterised by frequent reporting and sharing information on the course of treatment and its outcomes and good contact, was mentioned as most important by healthcare professionals (exp) to gain (more) trust in the MT. Healthcare professionals (exp and no-exp) indicated a shared vision of treatment and goalsetting as a prerequisite for professional collaboration.

*“When I refer a child*, *I would like to hear back how the treatment is going and what has been done*. *Open communication*, *where we respect each other*, *is really important for collaboration*.*” (H23*,*exp*)

#### Professional expertise

Whether parents and healthcare professionals (exp and no-exp) trusted a particular MT and would consider visiting or referring, depended on their experience with the MT’s professional competence and expertise. Some healthcare professionals and parents (exp) mentioned that they felt more trust once they had visited a treatment session and personally saw what the treatment entailed and how it was performed. PPTs and paediatricians (exp) considered MTs with additional education in treating infants and therefore registration in the Dutch registry for paediatric manual therapy, to be more reliable and trustworthy. The amount of experience with treating infants was mentioned as another important factor related to perceived professional expertise.

*“To me it is just really important that an MT is someone that I can trust*, *that he knows what he is doing and that I have seen him working*.*”(H03*,*exp*)

### Theme 4: Treatment experiences

#### Personal experiences

Parents (exp) mentioned that after manual therapy treatment they experienced reduced crying and restlessness in their infant and improvements in movements of the head. Parents (exp) indicated that because of positive experiences, they were more likely to consult the MT again or to positively advise other parents. Interviews revealed that these parents’ positive experiences could overrule negative attitudes and therefore directly influence the decision-making process to consult an MT. MTs and PPTs (exp) pointed out that they saw quick and positive results of treatment: the cervical mobility improved extensively. Although sometimes sceptical themselves, paediatricians (exp and no-exp) mentioned they heard parents’ positive experiences or observed improved mobility and reduced asymmetry themselves, which positively influenced their attitude.

*“Actually*, *it [treatment] helped very well with our daughter […]*. *Because we did not wait too long*, *symptoms were quite easy to resolve*.*” (P05*,*exp*)

Paediatricians (exp) who knew what the treatment entailed and had seen MTs treat infants, mentioned that, because of the quick and positive treatment outcomes, they were more likely to ignore the clinical practice guideline recommendation against referral. They expressed that instead they positively advised parents, but also pointed out they always let parents make their own decision. Given this, professional norms were overruled by personal experience.

*“I think it’s more about experience people have with manual therapy*. *If they do*, *[…] they know more about what they [manual therapists] do*. *I know that those colleagues ‘secretly’ refer people for manual therapy*. *Officially*, *according to the guideline*, *we have to discourage parents to go*.*” (H17*,*exp*)

#### Experiences from the social environment

Parents (exp and no-exp) stressed that experiences from friends and family were important to them. The social environment could scare them by telling there might be something wrong with their infant and they should consult an MT. These parents mentioned they sometimes felt pressured by the social environment. This phenomenon was also highlighted by healthcare professionals (exp and no-exp).

*“But I see in parents they fear something might be wrong*, *and feel*, *yes*, *how to say it*, *social pressure to seek for help*. *Parents tell each other they want the best for their child*, *really*, *they kinda push each other*.*” (H05*,*no-exp*)

### Theme 5: Emotions

Interviews revealed that emotions had a direct effect on parents’ decision-making and behaviour. Even when parents (exp) expressed a more negative attitude towards manual therapy, feelings of frustration, desperation and concern directly influenced their decision to consult an MT.

#### Feelings of frustration and desperation

Parents (exp) expressed they felt frustrated, helpless or desperate when their child cried excessively, had sleeping or feeding problems, seemed asymmetric or in pain. They felt that the stronger their emotions were, the more willing they were to try any treatment that might help their child.

*“I was so desperate*. *I didn’t know what to do anymore*. *I couldn’t handle it anymore*.*” (P07*,*exp*)

#### Feelings of concern

Due to crying, restlessness or skull deformation, parents (exp and no-exp) expressed concerns about their infant’s well-being, health, and skull and postural development. Parents (exp) felt an urge to seek help and highlighted they wanted to help their child so badly, that they therefore explored all possibilities. These parents believed they should just try it because it just might help. Parents’ feelings of concern were also mentioned by healthcare professionals (exp and no-exp).

*“I think that young parents can be really insecure and concerned; that makes them vulnerable*. *Their tendency to seek help and see every doctor or therapist because it might help*, *to stop the crying*, *is really strong*.*” (H04*,*exp*)

## Discussion

The online survey showed that MTs and PPTs were divided in two groups: those ‘in favour’ or ‘against’ manual therapy in infants. Reasons for not treating infants with manual therapy or no interprofessional collaboration were: limited knowledge, lack of professional competence or no personal contact with an MT, and negative beliefs regarding safety and effectiveness. Interviews enabled us to further explore underlying perspectives and attitudes and indicated that this distinction in two groups was also seen among in parents and other healthcare professionals. We found that attitudes were mainly impacted by knowledge and beliefs related to manual therapy’s scope, added value, and safety, professionals norms, interpersonal relation, and treatment experiences. Despite a positive or negative attitude, interpersonal relation, experiences, and emotions of frustration and desperation of parents were mentioned to have a direct impact on decision-making and choosing for manual therapy.

**Fig 2:**
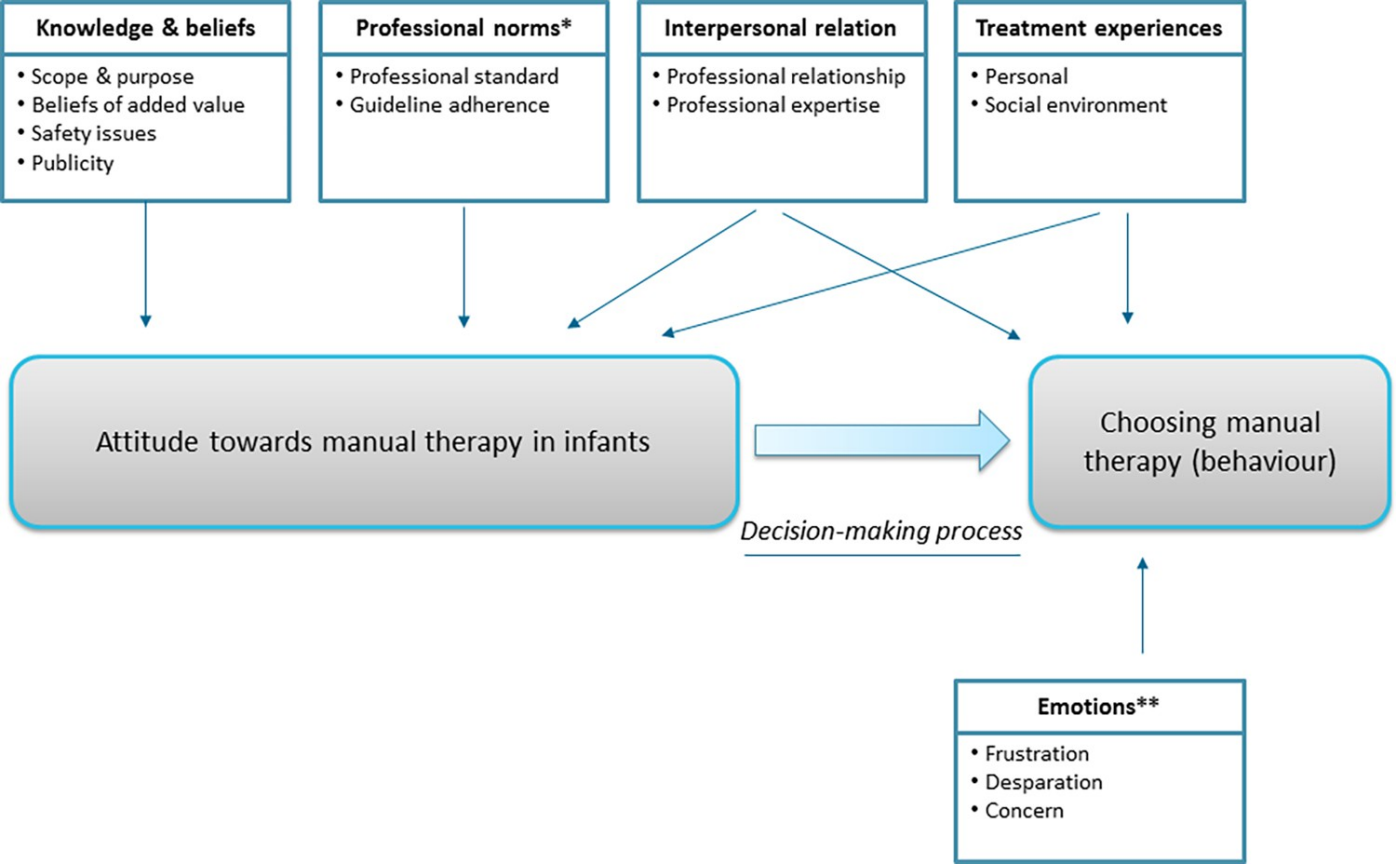
Conceptual model of factors related to attitude towards manual therapy in infants. *Factor that solely count for healthcare professionals, **factor that solely count for parents.

Factors that determine an individual’s attitude towards a particular concept and their related behaviour are frequently described in the field of health psychology. The Theory of Planned Behaviour, Social Cognitive Theory, and the Health Belief Model describe various determinants that, directly or indirectly, influence health-related behaviour [25]. To understand parents’ and healthcare professionals’ attitudes towards manual therapy in infants and related decision-making, we illustrated the explored determinants in a model (Fig 2). Parents and healthcare professionals indicated that knowledge and beliefs (partially influenced by media publicity), professional norms, treatment experiences, and interpersonal relation affected their attitude. Interpersonal relation, treatment experience and emotions of parents affected their behaviour directly, independent of their attitude. Parents and healthcare professionals explained that a good interpersonal relation with an MT affected feelings of trust and led to positive attitudes.

The importance of an interpersonal relation in physiotherapy was also highlighted in other studies [26–30]. Crom et al. showed that parents of paediatric patients reported two types of trust in the physiotherapist: trust in relational skills and trust in technical skills [27]. In addition, Peiris et al. showed that personal interaction with the physiotherapist was the main reason for positive experiences with physiotherapy care [30]. Parents and healthcare professionals in our study indicated positive treatment experience, personal or from the social environment, as important. Previous research on determinants of patient’s choice of healthcare providers showed similar results: personal experiences with a healthcare professional were most important and positively influenced the future choice for this particular professional [31]. Moreover, social norms, such as a professional’s reputation, recommendations from family and friends and a referral from their physician were indicated to have a strong positive effect on the choice for a particular healthcare professional [31]. In line with our study, the relationship showed to have a strong impact on the decision-making process, where a professional’s expertise and qualification and interpersonal factors, such as communication, empathy, and personal bond, were indicated as strong affecters [31]. With our study, we indicated that a good interpersonal relation with a manual physiotherapist and positive treatment experiences not only can affect the decision-making process of parents as healthcare seekers but also for healthcare professionals as referrers. Our findings even suggest that the impact of interpersonal relation and treatment experiences can be so strong, that it overrules negative attitudes based on professional norms or knowledge and beliefs, and can thereby directly affect the decision-making process on referral by healthcare professionals and choosing for manual therapy by parents. Moreover, since evidence on effectiveness of manual therapy in infants is lacking, the importance of clinical expertise, as described by Haynes [32], seems to increase. Decision-making mainly depends on treatment experiences and the professionalism and clinical expertise of a manual physiotherapist. These outcomes underline the importance of the personal aspect of healthcare and show that evidence, as described in guidelines, can be overruled. In particular in paediatric healthcare when parents can feel completely dependent on healthcare professionals for their child’s healthcare issues, factors as experience and trust might be important aspects in decision-making. Although manual physiotherapists differ in theoretical rationales and treatment techniques from other healthcare providers in the international field of manual therapy, such as chiropractors and osteopaths, the outcomes of our study can nevertheless be of added value for these areas in paediatric manual therapy.

### Strengths and limitations

A strength of this study was the mixed-methods design in which the early online survey prompted further in depth exploration of parents’ and healthcare professionals’ perspectives, underlying rationales and attitudes towards and experiences with manual therapy in infants. Another strength was the inclusion of a diverse population of parents and various healthcare professionals, with and without experience with manual therapy in infants. This enabled us to gain insight in the perspectives, attitudes and decision-making of a broad population. Selection bias may have occurred since participants with strong opinions, positive or negative, on this topic might have agreed to participate. The results of this study could therefore represent the stronger opinionated people. In addition, of all participants, parents were the hardest group to recruit. With our recruitment procedure we reached a fairly broad population as parents, and healthcare professionals, were equally distributed in those who had experience with manual therapy and those who did not. Furthermore, since a relatively small number of parents agreed to participate in this study, the results of this study are based on a small sample of parents (n = 7) compared to healthcare professionals (n = 23). Moreover, all included parents had higher education. However, an over representation of higher educated participants is consistent with previous studies on paediatric manual therapy, chiropractic, physiotherapeutic care, and complementary and alternative therapies [20, 33–36]. Possibly, higher educated people may more actively seek for conservative healthcare options, may have more access to these treatment options, and/or may be more willing to participate in scientific research.

## Conclusion

Parents’ and healthcare professionals’ attitudes towards manual therapy in infants can be divided as ‘in favour’ and ‘against’. Attitudes are affected by knowledge and beliefs, professional norms, treatment experiences and interpersonal relation. Despite lacking evidence on effectiveness, a positive treatment experience, a good interpersonal relation with a manual physiotherapist, and parental emotions of frustration and desperation can overrule negative attitudes and directly affect decision-making and choosing manual therapy.

## Supporting information

S1 Table Reasons for not collaborating and not treating infants. (DOCX)

S1 File Interview guide. (DOCX)
