# Supplementary material for: Parents’ and healthcare professionals’ perspectives on manual therapy in infants: A mixed-methods study
Source: PLoS One. 2023 Apr 6;18(4):e0283646. doi: 10.1371/journal.pone.0283646 (PMC10079100; doi:10.1371/journal.pone.0283646)
Supplement: S1 Table — (DOCX) [file pone.0283646.s001.docx]

**S1 Table. Reasons for not collaborating and not treating infants**

| **Reasons for no interprofessional collaboration** | **MTs (n=9)*** | **PPTs (n=91)** |
| --- | --- | --- |
| No (good) personal contact with other professional  No perceived added value  Lack of evidence for effectiveness  Policy of practice hinders collaboration  No interest in collaboration  No perceived treatment indication for manual therapy  Collaboration with osteopath instead of manual physiotherapist  Lack of competence (knowledge/skills)  Fear for complications | 3 (33%)  -  -  3 (33%)  3 (33%)  -  -  -  - | 10 (11%)  28 (31%)  16 (17%)  -  -  15 (16%)  8 (9%)  8 (9%)  6 (7%) |
| **Reasons for not treating infants** | **MTs (n=206)** | **PPTs (n=10)*** |
| Lack of professional expertise (knowledge, skills)  Infants are no target population of therapist or practice  Fear for complications  No perceived treatment indication for manual therapy  Lack of evidence for effectiveness  No experience  No perceived added value of therapy  No interprofessional collaboration | 47%  29%  6%  5%  5%  4%  2%  2% | 20%  80%  -  -  -  -  -  - |
| *MTs: manual physiotherapists, PPTs: paediatric physiotherapists*  **Not all manual physiotherapists and paediatric physiotherapists completed open-ended questions. Therefore, the amount of therapists differ from the total amount of therapists* | | |
