# Supplementary material for: Parents’ and healthcare professionals’ perspectives on manual therapy in infants: A mixed-methods study
Source: PLoS One. 2023 Apr 6;18(4):e0283646. doi: 10.1371/journal.pone.0283646 (PMC10079100; doi:10.1371/journal.pone.0283646)
Supplement: S1 File — (DOCX) [file pone.0283646.s002.docx]

**Supporting information**

**Interview guide**

Introduction

The aim of this study is to explore perceptions, opinions and experiences regarding manual therapy in infants. As described in the information letter you received, this interview will be audio-taped in order to guarantee I hear everything what you are telling me and will not miss information. Audiotapes will be processed anonymously. The interview will take about 30 minutes.

Do you still agree with audio taping?

In the Netherlands, many infants are treated by manual physiotherapists for asymmetry of the head or trunk. Often these infants cry excessively and are restless. In these infants, manual physiotherapists expect a dysfunction in the infant’s upper neck. However, there is no clarity about this concept. I would like to ask you some questions on your perceptions and potential experiences with manual therapy in these infants.

Please tell me when something is not clear to you during the interview.

Do you have questions?

**Topics for parents**

Manual therapy in general

- What are your thoughts according manual therapy in infants?
- What do you know about it?

Experience with manual therapy

- What is your experience with manual therapy in infants?
- What were your considerations to go/not go to the manual physiotherapist with your child?
- What made you decide to (not) go?
- How are you looking back at your decision?

**Topics for healthcare professionals**

Manual therapy in general

- What are your thoughts according to manual therapy in infants?

Experience with manual therapy

- What is your experience with manual therapy in infants?
- What are your considerations to (not) treat/refer these infants?
- What do you think about professional collaboration with manual physiotherapists?
